# Supplementary material for: When aggressiveness could be too risky: linking personality traits and predator response in superb fairy-wrens
Source: PeerJ. 2022 Sep 28;10:e14011. doi: 10.7717/peerj.14011 (PMC9526405; doi:10.7717/peerj.14011)
Supplement: Supplemental Information 2 — Model included (1) boldness (PC_Handling), (2) exploration (PC_Exploration), (3) aggressiveness (‘mirror attacks’, as a quadratic variable), (4) sex (male, female), (5) playback order (whether the predator playback was conducted first or second), and (6) number of responders (the number of group members that responded to the playback) as fixed factors and ‘Territory ID’ as a random effect. Statistically significant (≤ 0.05) values are marked in bold (N = 40 individuals). [file peerj-10-14011-s002.docx]

| **Fixed Effects** | **Estimate** | **Std. Error** | ***t*** | **P** |
| --- | --- | --- | --- | --- |
| *Model 3*  *Intercept* | -0.25 | 0.69 | -0.36 | 0.72 |
| Boldness | 0.10 | 0.15 | 0.68 | 0.50 |
| Exploration | -0.16 | 0.16 | -0.10 | 0.32 |
| Mirror attacks (continuous) | 0.10 | 0.05 | 2.03 | **0.05** |
| Mirror attacks (quadratic) | -0.003 | 0.001 | -1.97 | 0.06 |
| Sex | -0.38 | 0.30 | -1.24 | 0.22 |
| Playback order | 0.15 | 0.46 | 0.34 | 0.74 |
| Number of responders | 0.16 | 0.29 | 0.53 | 0.60 |
